# Lack of evidence of a beneficial effect of azathioprine in dogs treated with prednisolone for idiopathic immune-mediated hemolytic anemia: a retrospective cohort study

**DOI:** 10.1186/1746-6148-7-15

**Published:** 2011-04-13

**Authors:** Christine J Piek, Willem Evert van Spil, Greet Junius, Aldo Dekker

**Affiliations:** 1 Department of Clinical Sciences of Companion Animals, Utrecht, Utrecht University, PO Box 80154, 3508 TD Utrecht, The Netherlands; 2 Dierenkliniek Randstad, Frans Beirenslaan 155, 2150 Borsbeek, Belgium; 3 Central Institute for Animal Disease Control, P.O. Box 2004, 8203 AA Lelystad, The Netherlands

## Abstract

**Background:**

Azathioprine is used as an immunosuppressant in canine immune-mediated hemolytic anemia (IMHA), but this potentially toxic and carcinogenic drug has not been proven to be beneficial. The aim of this study was to determine the difference in outcome and survival of dogs with idiopathic IMHA treated with a protocol that included azathioprine and prednisolone versus a protocol that included prednisolone alone.

**Results:**

The study included 222 dogs with a hematocrit lower than 0.30 L/L and either a positive Coombs' test or spherocytosis and no evidence of diseases that could trigger IMHA. The clinical and laboratory data at the time of diagnosis and the response to therapy and survival were compared in dogs treated according to the prednisolone and azathioprine protocol (AP protocol; n = 149) and dogs treated according to the prednisolone protocol (P protocol; n = 73). At study entry, the two groups were comparable, except that thrombocyte counts were significantly lower and clinical signs had been present significantly longer in the AP protocol group. No significant difference in survival was found between the two groups: the 1-year survival was 64% (95% CI 54 - 77%) in the P protocol group and 69% (95% CI 59-80%) in the AP protocol group, respectively.

**Conclusions:**

Azathioprine would appear not to be beneficial as standard treatment for all cases of IMHA; however, a blinded, randomized clinical trial is needed to establish whether outcome is different with the two treatment protocols.

## Background

Glucocorticoids are the main component of the immunosuppressive treatment of canine idiopathic immune-mediated hemolytic anemia (IMHA), but cytotoxic drugs such as azathioprine are advised for severe disease, such as cases with intravascular hemolysis or autoagglutination, and if the disease is refractory to glucocorticoids alone [1-3]. A few studies have evaluated the effect of azathioprine in canine IMHA and reported conflicting results, ranging from a possible beneficial effect to no effect [4-7]. Cytotoxic drugs can have potentially severe side effects, such as bone marrow suppression [8] and gastrointestinal disturbances, and long-term adverse effects of cytotoxic drugs in humans and animals include neoplasms, leukemia, and testicular and ovarian dysfunction [9]. Similar problems have been reported in people working with these agents [9]. Azathioprine is recognized by the International Agency for Research on Cancer as being a possible or probable cancer-causing agent [9].

We previously reported on the treatment of dogs with idiopathic IMHA with an immunosuppressive protocol consisting of azathioprine and prednisolone, at that time the standard treatment in our clinic [10]. The lack of evidence of a beneficial effect of azathioprine in canine idiopathic IMHA combined with concerns about the safety of the drug with regard to animal owners, animal caretakers, and veterinarians prompted the question whether the use of azathioprine is justified in dogs with idiopathic IMHA. The standard treatment protocol was revised from one including combined therapy with azathioprine and prednisolone to one including prednisolone monotherapy. After several years, we now have sufficient documented cases to compare the efficacy of the two protocols. The aim of this study was to determine whether there are differences in the outcome and survival of dogs with idiopathic IMHA treated according to a protocol including azathioprine and prednisolone (AP) versus a protocol including prednisolone alone (P).

## Results

### Clinical characteristics of dogs

Of 108 dogs eligible for the P protocol, 32 met exclusion criteria and 3 further dogs were excluded because they switched from the P protocol to the AP protocol, leaving 73 dogs in the P protocol group. Of the 32 dogs excluded, 6 had been treated with glucocorticoids for more than 14 days, 5 had been diagnosed with babesiosis and/or ehrlichiosis (n = 2) or had visited an endemic area (n = 3), 8 had concurrent inflammatory disease (pneumonia n = 2, mesenteric lymphadenitis n = 1, gastroenteritis n = 3, dermatitis n = 1, necrotizing inflammation tail n = 1), 9 had neoplasia (carcinoma n = 3, hematopoietic tumors n = 5, hemangiosarcoma n = 1), and 3 had a concurrent immune-mediated disease (hypothyroidism n = 1, SLE n = 1, allergic dermatitis n = 1). The owner of 1 dog decided not to start treatment.

Of 197 dogs eligible for the AP protocol, 48 met exclusion criteria, leaving 149 dogs for inclusion in the AP protocol group, as described earlier [10]. Of the 48 dogs excluded, 10 dogs had been diagnosed with babesiosis and/or ehrlichiosis (n = 3) or had visited an endemic area (n = 7), 6 had concurrent lung disease, 4 had neoplasia (spleen n = 2, mediastinum n = 1, heart n = 1), 17 had hematopoietic tumors (myeloid leukemia n = 2, malignant lymphoma n = 8, malignant histiocytosis n = 5, hemangiosarcoma n = 2), 2 had SLE, and 1 had renal disease. Three dogs were treated with medications that can trigger IMHA, the owner of 1 dog chose not to start treatment, and data were incomplete for 4 dogs.

To exclude concomitant disease, the 73 dogs in the P protocol group underwent additional investigations, namely, thoracic radiography (n = 33), abdominal ultrasound (n = 52), cytological investigations (spleen n = 22, liver n = 31, lymph nodes n = 13, bone marrow n = 31, skin nodules n = 4), pathological examination (liver n = 2, spleen n = 1, intestinal biopsies n = 2), gastroduodenoscopy (n = 2), laparotomy (n = 1), electrocardiography (n = 1), and bacteriological investigations (n = 2). For the same reason, the 149 dogs included in the AP protocol group also underwent additional investigations, namely, thoracic radiography (n = 19), abdominal ultrasound (n = 69), cytological examination (spleen n = 9, liver n = 3, lymph nodes n = 4, bone marrow n = 17, skin nodules n = 1), pathological examination (liver n = 4, intestinal biopsies n = 2), gastroduodenoscopy (n = 2) dogs, explorative laparotomy (n = 1), electrocardiography (n = 2). Cytology of the spleen showed that the dogs in both groups had extramedullary erythropoiesis in the spleen and liver, and that most dogs had steroid-induced hepatopathy. There was no evidence of concomitant disease in any of the dogs.

The characteristics of the AP protocol group have been described earlier [10]. Of the 73 dogs that were included in the P protocol group, 12 dogs were crossbreeds, 5 were Maltese terriers, 4 were Jack Russell terriers, 4 were Labrador retrievers, 4 were Flat coated retrievers, 3 were English Springer spaniels, 3 were Dachshunds, 3 were Cairn terriers, 2 were Shetland sheepdogs, and 2 were Appenzeller Sennen dogs, respectively; the remaining 31 dogs were single dogs of other breeds. Twenty-seven of the 73 dogs were males (22 intact, 5 castrated) and 46 were females (24 intact, 22 castrated). In the AP protocol group, 61 were male (46 intact, 15 castrated) and 88 were female (51 intact, 37 castrated). There was no significant difference in the number of male and female dogs in the two groups (P = 0.77), or in the number of intact and neutered male and female dogs (P = 0.67). Median body weight at the time of diagnosis was 15.8 kg (range 2.5-45 kg) in the P protocol group and 18.7 kg (range 2.5-48.5 kg) in the AP protocol group (P = 0.44).

The median age at diagnosis of IMHA was 4.6 years (range 0.4 - 12.7 years) in the P protocol group and 5.7 years (range 0.3-13.9 years) in the AP protocol group; this difference was not statistically significant (P = 0.09). The median duration of clinical signs prior to diagnosis of idiopathic IMHA was 3 days (range 0-141 days) in the P protocol group and 6 (range 0-131 days) in the AP protocol group; this difference was significantly significant (P = 0.015). Anemia and clinical signs consistent with a tentative diagnosis of IMHA were documented by the referring veterinarian in 4 of 222 dogs 127, 128, 131, and 141 days before the diagnosis of idiopathic IMHA in our clinic.

There was no significant difference in the occurrence of anorexia, vomiting, diarrhea, red urine, dyspnea, fever, pale mucous membranes, icterus, or petechiae between the treatment groups. Information from the history and the physical examination, and the results of laboratory investigations at the time of diagnosis of IMHA are presented in Table 1. Thrombocyte counts were significantly lower in the AP protocol group than in the P protocol group at the time of diagnosis (P = 0.00001), but none of the other laboratory variables were significantly different at the time of diagnosis. The laboratory procedures for determining PT, APTT, fibrinogen, and thrombocytes changed during the study, but this led to significant differences in the results for PT and fibrinogen only. The mean PT before and after the change was 7.9 (n = 111) and 7.4 (n = 21) seconds (median 8 and 6.9 seconds), respectively, and the mean fibrinogen concentration before and after the change was 4.6 (n = 109) and 7.7 (n = 27) mg/L (median 4 and 7.5 mg/L), respectively. Only the PT and fibrinogen data for P protocol patients that entered the study before the change in laboratory procedures are included in Table 1 and were used in the univariate analysis. There were no significant differences in PT and fibrinogen levels between the two groups.

**Table 1:** Clinical signs and laboratory results for dogs in the AP protocol group and the P protocol group at time of diagnosis

	Azathioprine- Prednisolone Protocol				Prednisolone Protocol			
**Clinical signs**	**Present**	**Absent**	***n^a^***		**Present**	**Absent**	***n***	***P^b^***

Anorexia	119	30	149		55	17	72	0.554
Vomiting	44	105	149		27	46	73	0.263
Diarrhea	23	126	149		17	56	73	0.153
Dyspnea	16	133	149		4	69	73	0.199
Fever	69	80	149		33	38	72	0.981
Pale mucous membranes	146	3	149		72	1	73	0.735
Icterus	57	92	149		27	46	73	0.855
Petechiae	8	141	149		1	72	73	0.156
Red urine	47	102	149		17	55	72	0.223

**Laboratory results**	**Median**	**Range**	***n***	**Reference**	**Median**	**Range**	***n***	

Hematocrit (%)	13	0.04-0.27	149	42-57	12	0.05-0.26	73	0.36
Reticulocytes (%)	8	0.1-90	147	< 2	5.8	0.1-51.5	64	0.29
Corrected reticulocytes (%)	2.7	0.01-19.2	147	< 2	2	0.02-14.3	64	0.11
Osmotic red cell fragility (mOsm/L)	238	120-317	139	< 162	250	131-317	28	0.28
Leukocytes (x10^9^/L)	27.9	2.1-130	148	5.9-13.8	21.7	5.7-78.6	68	0.3
Band neutrophils (x10^9^/L)	1.4	0-22.1	148	0-0.3	1	0-21.9	67	0.08
Urea (mmol/L)	7.6	2.9-69.5	123	3.0-12.5	7.4	2.3-49.5	45	0.8
Creatinine (μmol/L)	41.8	0.4-652	112	< 50	41.2	6.5-228.4	48	0.16
PT^c ^(seconds)	8	6-12	98	7 ± 1	7	7-10	13	0.74
APTT^d ^(seconds)	19.8	11-98	98	14 ± 1	17.4	9.1-64.8	37	0.98
Fibrinogen (g/L)	3.9	0.6-13.8	96	2-5	5	2.3-8.2	13	0.11
Thrombocytes (x10^9^/L)	122	0-958	140	150-400	230	9-1079	65	0.00001*
Duration of clinical signs (days)	6	0-141	149		3	0-131	73	0.015*

^a ^Number of dogs in which the parameter was determined.

^b ^*P *= *P *value

^c ^PT = Prothrombin time

^d ^APTT = Activated partial thromboplastin time

### Therapy

Blood transfusions were given to 56 of 73 (76%) dogs in the P protocol group (once in 45 dogs, twice in 10 dogs, and three times in 1 dog) and in 98 of 149 (66%) dogs in the AP protocol group (once in 78 dogs, twice in 18 dogs, three times in 1 dog, and four times in 1 dog). The difference in transfusion requirement between the two groups was not statistically significant (P = 0.61). The median duration of prednisolone therapy was 68 days (range 0-936; n = 64) in the P protocol group and 59 days (range 0-622; n = 92) in the AP protocol group; the difference in treatment duration was not statistically significant (P = 0.20). In the AP protocol group, the median duration of azathioprine therapy was 53 days (range 0-622; n = 83).

### Outcome

Dogs in the P protocol group made the first return visit to the clinic after a median of 17 days (range 0-141; n = 43) and the second return visit after a median of 56 days (range 21-171; n = 25). Dogs in the AP protocol group made the first return visit to the clinic after a median of 25 days (range 2-83; n = 95) and the second return visit after a median of 77 days (range 21-399; n = 69). The first and second return visits were significantly earlier in the P protocol group than in the AP protocol group (P = 0.001 and 0.0032, respectively, Table 2). Apart from a significantly higher reticulocyte count in the P protocol group at the first return visit (P = 0.027), there were no significant differences in hematocrit, thrombocytes, and red cell osmotic fragility between the two groups at either visit (Table 2). Eight dogs in the P protocol group relapsed after a median of 63 days (range 7-581 days), as did 17 dogs in the AP protocol group after a median of 112 days (range 32-1757 days). There was no significant difference in the time to relapse (P = 0.277) or in the number of relapses (P = 0.8939) between the two groups.

**Table 2:** Results for laboratory tests and response categories for dogs with idiopathic IMHA treated according to the AP protocol (n = 149) or the P protocol (n = 73) group at the time of the first and second return visits and relapse

	Azathioprine-Prednisolone Protocol				Prednisolone Protocol			
**First return visit**	**Median**	**Range**	***n***	**Reference**	**Median**	**Range**	***n***	***P^b^***

Hematocrit (%)	0.35	0.06-0.5	93	42-57	0.36	0.12-0.47	41	0.5
Thrombocytes (x10^9^/L)	402	8-986	88	150-400	421	20-1555	29	0.48
Reticulocytes (%)	0.9	0.1-28	87	< 2	2	0.2-6.4	34	0.027*
Osmotic red cell fragility (mOsm/L)	176	136-258	82	< 162	164	125-247	24	0.06
Time after diagnosis ^g ^(days)	25	2-83	95		17	0-141	43	0.001*
								
**Response category^a^**	**No. dogs**		***n***		**No. dogs**		***n***	***P^b^***
No response^c^	3		95		1		26	
Improvement^d^	87		95		25		26	
Complete recovery^e^	5		95		0		26	0.92

**Second return visit**	**Median**	**Range**	***n***	**Reference**	**Median**	**Range**	***n***	***P^b^***

Hematocrit (%)	0.4	0.11-0.54	66	42-57	0.42	0.18-0.51	24	0.38
Thrombocytes (x10^9^/L)	278	3-834	63	150-400	339	12-636	17	0.73
Reticulocytes (%)	0.8	0.1-12	60	< 2	1	0.3-9.7	18	0.39
Osmotic red cell fragility (mOsm/L)	163	133-238	58	< 162	165	142-206	11	0.40
Time after diagnosis ^g ^(days)	77	21-399	69		56	21-171	25	0.0032*

**Response category^a^**	**No. dogs**		***n***		**No. dogs**		***n***	***P^b^***
No response	0		62		0		13	
Improvement	42		62		12		13	
Complete recovery	20		62		1		13	0.12

**Relapse**^f^	**Median**	**Range**	***n***	**Reference**	**Median**	**Range**	***n***	***p^b^***

Hematocrit (%)	0.28	0.06-0.44	17	42-57	0.31	0.10-0.41	5	0.96
Thrombocytes (x10^9^/L)	132	0-382	15	150-400	327	228-426	2	0.15
Reticulocytes (%)	2.5	0.1-22	14	< 2	0.3	0.2-30.5	3	0.9
Osmotic red cell fragility (mOsm/L)	212	159-274	12	< 162	221	187-254	2	0.65
Time after diagnosis^g ^(days)	112	32-1757	21		63	7-581	8	0.27

^a^Number of dogs counted in each response category.

^b ^*P *= P value

^c ^No effect of therapy was defined as no increase in the hematocrit.

^d ^Improvement was defined as an increase in hematocrit to < 0.36 L/L, or a hematocrit >0.36 L/L but with a positive Coombs' test or an increased osmotic red cell fragility.

^e ^Complete recovery was defined as an increase in the hematocrit to >0.36 L/L, a negative Coombs' test, and an osmotic red cell fragility within the reference range.

^f ^Relapse was defined as a decrease in the hematocrit after an initial improvement or complete recovery in combination with the recurrence of a positive Coombs' test or increased red cell fragility.

^g ^The times of the first and second return visits or when a relapse occurred were retrieved from the medical record.

### Analysis of survival

**Figure 1:**
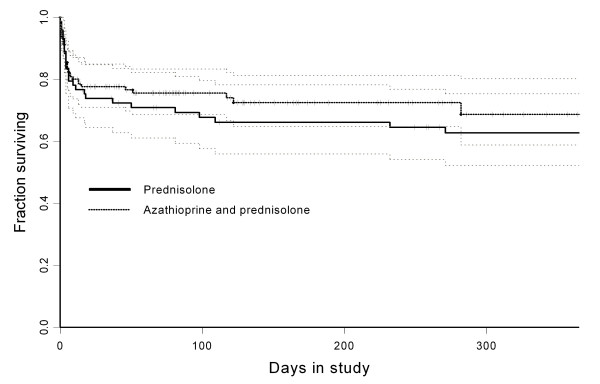
**Kaplan-Meier survival curve for dogs with idiopathic IMHA treated according to the AP protocol (n = 149) or the P protocol (n = 73)**.

The 1-year survival was 64% (95% CI, 54-77%) in the P protocol group and 69% (95% CI, 59-80%) in the AP protocol group, respectively (Figure 1); this difference in survival was not statistically significant (P = 0.65). The difference in survival at 14 days, 6 months, and 1 year was 1.7% (95% CI, minus 10 - 14%), 6.4% (95% CI, minus 7.4 - 20%), and 6.4% (minus 11 - 19.9%), respectively. At 1 year, 25 dogs had died of IMHA and 13 dogs were censored (1 had died of other causes, 9 were alive with treatment, and 3 were alive without treatment) in the P protocol group, and 36 dogs had died of IMHA and 106 dogs were censored (3 had died of other causes, 74 were alive with treatment, and 26 were alive without treatment) in the AP protocol group. Survival among dogs that lived longer than 14 days (n = 151) was not significantly different between the two groups (P = 0.649)(Table 3).

**Table 3:** Life table and estimated survival results for the AP protocol group (n = 149) and P protocol group (n = 73) at 14 days, 6 months, and 1 year after the date of diagnosis of idiopathic IMHA and at the time of the scheduled first visit (28 days) and second control visit (70 days)

Azathioprine-Prednisolone Protocol					
**Time (days)**	**# at risk^a^**	**Cumulative** **# events^b^**	**Cumulative** **# censored^c^**	**Estimated survival**	**95% CI** **Interval**

14	98	30	21	0.785	0.719 - 0.856
28	61	31	57	0.776	0.710 - 0.849
70	67	32	50	0.756	0.686 - 0.833
182	31	35	86	0.725	0.647 - 0.812
365	12	36	106	0.685	0.588 - 0.802
					
**Prednisolone Protocol**					

14	56	17	0	0.767	0.676 - 0.870
28	53	19	1	0.738	0.644 - 0.847
70	46	21	8	0.709	0.611 - 0.822
182	42	24	11	0.661	0.559 - 0.782
365	36	26	18	0.643	0.539 - 0.767

^a ^Number of dogs alive.

^b ^Cumulative number of dogs in each protocol group that died of idiopathic IMHA.

^c ^Cumulative number of dogs in each protocol group that were censored.

The proportional hazards assumption was valid for all variables that were significant in univariate and multivariate analyses. Results of the univariate analysis of the pooled data from the two groups with P < 0.20 and the variable treatment (P = 0.65) are presented in Table 4. The best multivariate model included plasma urea concentration (HR = 2.56; 95% CI, 1.729-3.789; n = 164) and icterus (HR = 2.94; 95% CI, 1.60-5.42; n = 164) as positive predictors of death, and spherocytes (HR = 0.38; 95% CI, 0.2-0.72; n = 164) as a negative predictor of death. The HRs were calculated for clinically relevant intervals.

**Table 4:** Univariate and multivariate Cox proportional hazards results for risk of death in 222 dogs with idiopathic IMHA for variables determined at time of first diagnosis with P < 0.20 and the variable "treatment protocol"

	Univariate analysis			
**Variable^a^**	**Hazard ratio**	***N*^b^**	**95% CI**	***P***^c^

Icterus	2.47	222	1.52 - 4	0.0003*
Urea (20 mmol/L)	2.22	168	1.55-3.19	0.0004*
Creatinine ^d ^(50 μmol/L)	1.28	160	1.15-1.42	0.0012*
Red cell osmotic fragility	0.426	181	0.221 - 0.819	0.018*
Thrombocytes (50 ×10^9^/L)	0.902	205	0.821-0.991	0.0184*
Age (years)	1.09	221	1.01-1.17	0.0266*
APTT^e ^(seconds)	1.03	135	1-1.05	0.0445*
Spherocytes	0.663	215	0.396 - 1.11	0.115
Hematocrit (L/L)	0.0173	222	0.00011 - 2.77	0.109
Treatment protocol	1.12	222	0.679-1.86	0.65
				
	**Multivariate analysis**			

Urea (20 mmol/L)	2.56	164	1.729-3.789	0.0001*
Icterus	2.94	164	1.60 - 5.42	0.0005*
Spherocytes	0.38	164	0.2 - 0.72	0.0023*

^a ^Variables were entered in the Cox proportional hazards model either as a factor or as a continuous variable, in which case the hazard ratio was calculated for the interval that is given in the table.

^b ^Number of dogs is given in which the parameter was determined for which the Cox proportional hazards model calculated the hazard ratio.

^c ^*P *= P value

^d ^Plasma creatinine concentrations were corrected for body weight (van den Brom and Biewenga, 1981).

^e ^APPT = Activated partial thromboplastin time

## Discussion

The aim of this study was to determine whether treatment according to a protocol including azathioprine and prednisolone (AP) compared with a protocol including prednisolone (P) alone leads to differences in outcome and survival in dogs with idiopathic IMHA. There were no significant differences between the treatment groups in the duration of immunosuppressive therapy, number of blood transfusions, survival (Figure 1), or treatment response.

Thrombocyte counts at the time of diagnosis were significantly lower in the AP protocol group than in the P protocol group. Although a low thrombocyte count has a negative influence, mainly on short-term survival [10,11], it is unlikely that the lower thrombocyte count in the AP protocol group masked a potential beneficial effect of azathioprine for a number of reasons. Firstly, the most likely explanation for the low thrombocyte count in dogs with IMHA is the decrease over time due to both immune-mediated destruction and thrombotic tendencies [11-14]. The median duration of clinical signs prior to diagnosis of idiopathic IMHA was the longest in the AP protocol group, which might explain the lower thrombocyte count in this group. Studies of IMHA show survival curves with similar slopes, with most deaths occurring in the first 2 weeks after diagnosis, despite differences in severity of clinical disease [4,5,15]. This suggests that recovery from the acute IMHA crisis and the associated pathology, such as thrombosis and disseminated intravascular coagulation, takes about 2 weeks. Indeed, at the first return visit thrombocyte counts were no longer different between the two groups (Table 1). We have previously found that thrombocytopenia at the start of therapy decreases short-term survival but not long-term survival [10]. On the basis of this, it is unlikely that the difference in thrombocyte count at the time of diagnosis had an effect on long-term outcome. Secondly, it is debatable whether azathioprine has a clinical effect within 2 weeks of therapy initiation in dogs. Although azathioprine decreased the lymphocyte blastogenic response in dogs after 7 days of treatment [16], it induced significant changes in immunoglobulin levels and lymphocyte numbers only after 2 weeks of treatment [17]. Thirdly, the best multivariate Cox proportional hazards model (Table 3) contained urea plasma concentration, icterus, and spherocytes as significant predictors of death due to IMHA. Neither inclusion of the variable "treatment protocol" nor "thrombocytes" improved this model significantly. For these reasons, we conclude that the difference in thrombocyte count between the two protocol groups at the start of the study is not likely to have had an influence on long-term survival.

One of the drawbacks of using historical controls is that time-related differences in the study population or in the treatment received, other than the effect of azathioprine, might confound the effect of treatment. Because inclusion criteria were unchanged throughout the trial and based on the results of objective quantitative laboratory tests, it is unlikely that this led to unrecognized inclusion bias. To minimize exclusion bias based on time-related differences in the diagnostic work-up, at the time of the study each individual case was evaluated by one of the authors to ensure that causes of secondary IMHA had been appropriately excluded. Although the addition of azathioprine was the only identifiable difference in treatment between the two groups, it cannot be excluded that supportive care in the intensive care unit had improved during the trial. In our institute, contrary to what is advocated by others [7], it is not routine practice to use antithrombotics or anticoagulants that might otherwise have influenced outcome.

Although there were no treatment-related differences in treatment response and survival, there were some differences between the laboratory results at the time of first and second return visit. The reticulocyte count in the P protocol group was significantly higher, which, although modest, might indicate that the red cell regeneration response was still active, because the first return visit in the P protocol group was 6 days earlier than that of the AP protocol group. Alternatively, it might reflect azathioprine-induced bone marrow suppression in the AP protocol group [8]. Azathioprine related side effects were noted in 12 of 149 (8.1%) dogs in the AP protocol group [10]. This seems a less likely explanation, however, since hematocrit, leukocyte, and thrombocyte counts at the time of first return visit were not significantly different between the two treatment groups. Eighteen dogs in our study developed idiopathic IMHA before the 1 year of age, in contrast with previous reports describing an onset only after the first year of age [3-5,15,18]. The clinical and laboratory findings and survival of these 18 dogs were not significantly different from those of the other 204 dogs in this study (data not shown).

Azathioprine is listed as a human carcinogen [9], and for this reason its use should be restricted in veterinary medicine to indications for which an evidence-based effect has been demonstrated, or to studies seeking to prove its beneficial effect [9]. While there was no significant difference in 1-year survival between the two groups, the confidence interval included both a 20% superior survival and a 11% lower survival. Given the limitations that are inherent to a retrospective study, this potential difference in outcome might be regarded as clinically significant. A randomized placebo-controlled study is necessary to estimate the true effect size of the AP protocol for the treatment of canine IMHA. The estimated effect size in this study can be used for sample size calculations [19]. However, given the side effects in dogs and the carcinogenicity in humans, we feel that the findings of this study do not justify the use of azathioprine in each IMHA patient. Given that at least 95% of the dogs in both protocol groups were classified as improved or completely recovered at the first control visit a median of 25 days after the start of therapy (Table 2), we suggest that the addition of azathioprine to the prednisolone protocol should be considered if there is no, or an inadequate, response to prednisolone after 2-3 weeks of treatment, provided the guidelines for adjustment of the prednisolone treatment have been followed.

## Conclusions

The absence of a statistically significant difference in survival indicates that there may be no beneficial effect of including azathioprine in the standard treatment of all cases of IMHA; however a blinded, randomized clinical trial is needed to establish the true difference in effect between the two treatment protocols.

## Methods

### Patients

The dogs had been referred to the Utrecht University Clinic of Companion Animals (UUCCA) from 1st January 1994 to 31st December 2005. Inclusion criteria were a hematocrit < 0.30 L/L and either a positive Coombs' test or the presence of spherocytes in a blood smear. All dogs had been treated according to either a standard immunosuppressive protocol consisting of azathioprine and prednisolone (1st January 1994 until 31st December 2000) or a protocol consisting of prednisolone alone (1st January 2002 until 31st December 2005). A complete medical record had to be present.

Dogs were excluded if they had evidence of diseases that could induce IMHA, such as neoplasia, medications, and infectious diseases. As a result, dogs that had visited areas where ehrlichiosis and babesiosis are endemic within 3 weeks of the diagnosis of idiopathic IMHA were excluded unless serologic examination for *Ehrlichia canis *and *Babesia canis *or *B. gibsonii *was negative. Dogs that had received immunosuppressive treatment for longer than 14 days before referral to the UUCCA were excluded. Dogs that were referred between 31st December 2000 and 1st January 2002 were excluded to avoid possible selection bias, since in that period both treatment protocols were used.

Breed, sex, complete history, and physical examination were recorded, including age at time of first diagnosis of idiopathic IMHA, anorexia, vomiting, diarrhea and dark red urine, as well as the presence of an increased rectal temperature, pale mucous membranes, icterus, and petechiae. Additional diagnostics were performed if judged necessary by the attending clinician.

### Laboratory tests

All tests at the time of diagnosis were performed at the UUCCA. The outcomes of the following laboratory tests performed on admission were retrieved from the medical records: complete blood count (CBC), reticulocyte count, presence of spherocytes (confirmed by one of the authors, CP), Coombs' test, osmotic red cell fragility, plasma urea and creatinine concentrations. Data that were available for a subset of the patients only were not recorded. During return visits, approximately 4 and 10 weeks after start of treatment, CBC, reticulocyte count, Coombs' test and osmotic red cell fragility were determined. A monovalent direct Coombs' antiglobulin test was performed using anti-dog IgG (Central Blood Laboratory, Nordic), anti-dog IgM antibodies (Nordic) and, before January 2005, an anti-dog complement antibody (Nordic), for agglutination of the patients' red cells; results were reported as negative, weakly positive, or positive. The osmotic fragility of erythrocytes was determined as previously described [20]. Plasma creatinine concentrations were corrected for body weight [21].

The prothrombin time (PT), activated partial thromboplastin time (APTT), and fibrinogen concentration were recorded when measured (Option 4, BioMerieux). PT and APTT were considered prolonged if they were increased at least 10% above levels measured in normal pooled canine citrated plasma. From March 2003 onward, coagulation profiles were determined with the Thrombolyser Compact-X (Trinity Biotech PLC) and new reference values were established for this system. From March 2003 onward, the hematology analyzer was changed from the combination of the ABX Helios and ABX Helios 5 Diff (ABX International) to the ADVIA 120 (Siemens Medical Solutions Diagnostics) and reference values were established for this system.

### Therapy

Blood transfusions, alone or in combination with IV fluid therapy, were given if considered necessary by the attending clinician. A transfusion consisted of the equivalent of 450 mL of donor blood. In dogs weighing less than 10 kg, a lower transfusion volume was given, with a maximum of 40 mL/kg. Depending on availability, either packed red blood cells or fresh whole blood was used. The number of blood transfusions was noted. All clinic-owned donor dogs used in the study were DEA 1.1 and 1.2 negative. No cross-match was performed in the case of first blood transfusion. Only in the case of client-owned donor dogs or if a second transfusion was given, were cross matches performed.

Two treatment protocols were used. A standard protocol consisting of a combination of prednisolone and azathioprine (AP protocol) was instituted in dogs that were treated before 31st December 2000. From 1st January 2002 onward, the standard protocol for dogs with idiopathic IMHA contained prednisolone only (P protocol). In both protocols prednisolone was given following the same schedule. The response category (see below) was assessed at least once daily during hospitalization and during return visits that were scheduled 4 and 10 weeks after the start of therapy. As long as the response category was no change, prednisolone (Alfasan) was given in a dosage of 2 mg/kg/day PO. Dogs that were not able to take oral medication were hospitalized and treated with dexamethasone (0.5-1 mg/kg/day) IV or SC. Once the response had improved, prednisolone therapy was started at a dosage of 2 mg/kg/day PO for 3 days, followed by 1.5 mg/kg/day PO for 7 days, 1 mg/kg/day PO for 10 days, 0.5 mg/kg/day PO for 14 days, after which the same dose was given on alternate days for 14 days and subsequently tapered down to 0.25 mg/kg/day PO for 21 days. If the outcome was assessed as complete recovery at the 4- or 10-week return visit, the prednisolone therapy protocol described above was followed. If relapse occurred at any time during this treatment, the prednisolone therapy protocol was started from the beginning. If at the 4 or 10 week return visit the treatment response was assessed as improved but there was no complete recovery, the duration of the intervals during which prednisolone was tapered, as described above, was doubled. Additionally, in the AP protocol, dogs were treated with azathioprine (Imuran, Glaxo-Wellcome) at a dosage of 2 mg/kg/day PO for dogs weighing < 20 kg. The daily azathioprine dose in dogs of 25, 30, 40 and 50 kg was maximized at 45, 50, 60 and 70 mg, respectively. Azathioprine treatment was stopped 10 days after prednisolone treatment.

### Outcome

The response to therapy was retrieved from the medical record. The response was assessed at the actual time of the first and second return visits. At both times the findings were compared to those of the last visit. We defined four response categories. Complete recovery was defined as an increase in the hematocrit to >0.36 L/L, a negative Coombs' test, and an osmotic red cell fragility within the reference range. Improvement was defined as a modest increase in the hematocrit or an increase to >0.36 L/L but with a positive Coombs' test or an increased osmotic red cell fragility. No response was defined as no increase in the hematocrit. And relapse was defined as a decrease in the hematocrit after an initial improvement or complete recovery in combination with the recurrence of a positive Coombs' test or increased red cell fragility. The time at which a relapse occurred was retrieved from the medical record.

Survival was determined by telephone contact with the owner by one of the investigators (GJ for the AP protocol group and ES for the P protocol group). If we were unsuccessful in contacting the owner, the last date of contact was recorded from the file. The outcome at the last date of contact was divided into three categories: death due to IMHA, death due to another cause, and alive with or without treatment.

### Statistical analysis

Statistical analysis of the data was performed using S-plus statistical package (Insightful Corporation). Comparisons between the two protocol groups were made assuming no difference between the treatment groups. Non-parametric tests were used when the data were not normally distributed (Chi-square or Fisher's exact test for categorical variables and Two-sample Wilcoxon rank-sum test for continuous variables).

As described above, minor changes were made in the laboratory procedures for PT, APTT, fibrinogen, and thrombocytes during the period that dogs were entered in the P protocol group. The results for these variables in the P protocol group were split into two groups, before and after the change, and handled as individual groups during the statistical analysis. Comparisons between the AP protocol group and both subgroups of the P protocol groups were made using the Kruskall-Wallis χ^2 ^test. In case of multiple testing, Bonferroni correction was performed.

Survival analysis was performed for the AP protocol group and the P protocol group. The end point was death due to IMHA. Dogs that were alive at the end of the study or had died of other causes were censored. Survival curves were drawn with the Kaplan Meier method.

The treatment protocol used and the variables recorded at the date of first diagnosis, with the exception of the variable breed, were evaluated in a univariate Cox proportional hazard model and used to generate hazard ratios (HRs). The variable treatment protocol and the variables significant at the P < 0.20 level in the univariate analysis were introduced in a multivariate model, allowing for interaction between variables. Multivariate analysis was performed by forward stepwise selection using a probability of P < 0.05 in the likelihood ratio test as a criterion for inclusion. Compliance with the proportional hazards assumption was tested graphically by plotting the Schoenfeld residuals against time.

## Authors' contributions

CJ collected the clinical and laboratory data, performed the statistical analysis and drafted the manuscript. ES participated in the collection of the clinical and laboratory data, the design and the statistical analysis of the study. GJ participated in the collection of the clinical and laboratory data and the design of the study. AD performed the statistical analysis and participated in the design of the manuscript. All authors read and approved the final manuscript.
